# Network analysis of depression and anxiety symptoms and their associations with life satisfaction among Chinese hypertensive older adults: a cross-sectional study

**DOI:** 10.3389/fpubh.2024.1370359

**Published:** 2024-03-18

**Authors:** Hongfei Ma, Meng Zhao, Yangyang Liu, Pingmin Wei

**Affiliations:** Department of Epidemiology and Health Statistics, School of Public Health, Southeast University, Nanjing, Jiangsu, China

**Keywords:** hypertensive older adults, network analysis, depression, anxiety, life satisfaction

## Abstract

**Background:**

Hypertension is one of the most prevalent chronic diseases among the older adult population in China and older adults with hypertension are more susceptible to mental health problems. This study aimed to explore the network structure of depression and anxiety, and their association with life satisfaction (LS) in older adults with hypertension.

**Methods:**

A total of 4,993 hypertensive individuals aged 60 and above were selected from the Chinese Longitudinal Healthy Longevity Survey (CLHLS 2017–2018). The design of the CLHLS study was approved by the Campus Institutional Review Board of Duke University (Pro00062871) and the Biomedical Ethics Committee of Peking University (IRB00001052-13,074). The Center for Epidemiologic Studies Depression Scale-10 (CESD-10) and the Generalized Anxiety Disorder Scale-7 (GAD-7) were used to assess depressive and anxiety symptoms. Central and bridge symptoms were identified via “Expected Influence” and “Bridge Expected Influence”, respectively. Network stability was assessed using the case-dropping bootstrap technique.

**Results:**

Network analysis identified CESD3 (Feeling blue/depressed), GAD4 (Trouble relaxing), and GAD2 (Uncontrollable worry) as the most influential central symptoms in the network of depression and anxiety. Concurrently, GAD1 (Nervousness or anxiety), CESD10 (Sleep disturbances), and CESD1 (Feeling bothered) stand as critical bridge symptoms between depression and anxiety disorders. Moreover, CESD7 (Lack of happiness) exhibited the strongest negative correlation with LS in Chinese hypertensive older adults.

**Conclusion:**

This exploratory study represents the first investigation to examine the mutual relationship between depressive and anxiety symptoms among Chinese hypertensive older adults. Interventions addressing targeting bridge symptoms have the potential to alleviate depressive and anxiety symptoms. Furthermore, improving happiness, hope, and sleep quality in this population may mitigate the adverse effects of depression and anxiety on LS.

## 1 Introduction

As life expectancy increases and birth rates decline, the proportion of older adult individuals (aged ≥60 years) continues to grow within the global population (1). China also has a significant older adult population. According to China’s seventh population census, the older adult population in China was 264 million in 2021, comprising 18.7% of the country’s total population (2). Furthermore, China’s aging burden will intensify as the second wave of baby boomers (those born between 1962 and 1975) begins to retire in 2022 (3). Hypertension ranks among the most prevalent chronic conditions among the older adult population in China. A study investigating seven chronic diseases among older adults in Chinese communities revealed hypertension as the most prevalent condition, with 50% of the surveyed participants reporting hypertensive disorders (4). Older adults with hypertension inherently have a more delicate constitution compared to their healthier counterparts. If they fail to maintain regular antihypertensive treatment, they are more prone to severe complications such as coronary heart disease, heart failure, cerebral infarction, or cerebral hemorrhage (5). The physical complications and uncertainty about future health could potentially impose substantial psychological stress on older adults afflicted with hypertension, leading them to grapple with psychological afflictions. A comprehensive review conducted in 2021 confirmed that older adults with hypertension are more vulnerable to mental health disorders including depression, anxiety, loneliness, and apprehension (6).

Multiple scholarly investigations have indicated a significant correlation between hypertensive disorders and symptoms associated with depression, with patients diagnosed with hypertension being at a heightened risk of developing depressive symptoms (7–9). Patients suffering from hypertension are prone to physical symptoms such as dizziness, headaches, chest tightness, reduced sleep quality, and decreased quality of life which may affect their psychological state and lead to depression (10). Many individuals diagnosed with hypertension require lifelong medication. Failure to take medication regularly can easily exacerbate the severity of physical symptoms and may lead to complications of hypertension, which can cause some psychological stress and negative emotions in the patients (5, 9). Moreover, the physiological changes and increased emotional vulnerability associated with the aging process in the older adults, along with the influence of hypertension, contribute to a higher prevalence of depression (9). According to previous study, anxiety disorders are also prevalent psychological disorders in the older adult population, and older adults with anxiety disorders may simultaneously suffer from depression (11). The survey conducted in South Korea involving 1,204 community-dwelling older adults revealed that 275 individuals (22.8%) suffered from comorbid anxiety and depression (11). Moreover, anxiety disorders are highly prevalent in individuals with hypertension, and they are closely associated with depression (12). The study conducted by Zhang among hospitalized hypertension patients in China confirmed the co-morbidity of depression and anxiety in the hypertensive population (12). Due to the significant overlap between the risk factors associated with hypertension and those connected to anxiety and depression, both two psychological disorders frequently co-occur in hypertensive patients, highlighting the complex interplay between mental health and cardiovascular health (12, 13). However, previous studies have predominantly relied on global scores to evaluate the comorbidity of depression and anxiety in older adults with hypertension, which fails to capture the interplay between symptoms of the two disorders.

Network models in psychological research suggest that the exacerbation of one symptom may lead to the exacerbation of other related symptoms, thereby triggering a chain reaction (14). Similarly, the alleviation of one symptom may have a positive effect on other symptoms (15). The counterpart of the network model is the network analysis method, which quantifies the relationship between individual symptoms of mental disorders (16). On one hand, network analysis can estimate the interconnectedness among the symptoms of the disease itself and identify closely related symptom groups (14). On the other hand, it may reveal central symptoms (core symptoms) that contribute to the development and/or maintenance of the psychopathological network (17). Central symptoms, which may have a greater impact on the system than peripheral symptoms, are thought to contribute to the rapid activation of interrelated symptoms within the network and may be targeted for treatment (18). Additionally, network analysis can also identify bridge symptoms between different categories of psychiatric disorders (19). Interventions targeting bridge symptoms may lead to a significant reduction in the association between diseases, thereby further reducing the incidence of comorbidities.

In previous studies, network analysis methods have been employed to investigate the relationship between symptoms of depression and anxiety disorders among populations with different cultural backgrounds and diverse professions. For instance, these studies have examined populations such as clinical healthcare workers (20, 21), hospitalized patients in Germany (22), adolescents in India (23), and disabled older adults in China (19). Furthermore, researchers have investigated the network structure and bridge symptoms of depression and anxiety in populations affected by specific conditions (24–26). Chen conducted a network analysis study in 566 tinnitus patients and found the presence of six robust connections and three bridge symptoms in the depression-anxiety network (24). However, the results of the network analysis of depression and anxiety in the epilepsy sample and psychiatric sample differed from those of the tinnitus patients (25, 26). The variation in network analysis outcomes across prior studies highlighted the substantial dissimilarities in results among diverse populations, making it inappropriate to generalize findings across populations (23). In China, there is a substantial and rapidly expanding population of older adults affected by hypertension, who are considered highly susceptible to depression and anxiety disorders (3, 4). Nevertheless, the relationship between particular symptoms of depression and anxiety in the hypertensive older adult population remains unclear and has not been investigated thus far. Additionally, individual life satisfaction (LS), which is a facet of subjective well-being, exhibited a negative correlation with individual depression and anxiety (27). A study conducted in Germany, examining the depression, anxiety, and life satisfaction of parents caring for children with cystic fibrosis, discovered that higher levels of depression and anxiety correlated with lower levels of LS (28). The interaction between specific symptoms of depression and anxiety with LS remains somewhat ambiguous, highlighting the need for further investigation into this research inquiry.

The older adult population with hypertension is more likely to encounter an increased array of health challenges and limitations due to the age increase, which can potentially exacerbate the severity of their own depression and anxiety symptoms (6). Unfortunately, very few scholars have utilized network analysis as a data analysis approach to explore the relationship between depression, anxiety, and life satisfaction among older adults with hypertension in China from the perspective of symptoms. This scarcity of research served as the motivation for conducting our study. The objective of this study was to examine the network characteristics of depression and anxiety in hypertensive older adults in China, while also exploring the links between LS and depressive and anxiety symptoms.

## 2 Methods

### 2.1 Participants and procedures

Data from the 7th wave (2017–2018) of the Chinese Longitudinal Healthy Longevity Survey (CLHLS) was selected for our study. The CLHLS project is organized by the Center for Healthy Aging and Development Studies at Peking University. This project has conducted longitudinal surveys in 23 provinces of China in seven waves [2000, 2002, 2005, 2008–2009, 2011–2012, 2014, and 2017–2018] (29). The CLHLS adopted a multi-stage disproportionate and targeted random sampling method to ensure a representative sample, focusing on individuals aged 60 and above (15).

Participants included in this study were older adults (aged ≥60 years) diagnosed with hypertension at the hospital. After excluding questionnaires with incomplete information, the study included a sample size of 4,993. During the field survey, all hypertensive older adults signed an informed consent form under the direction of the investigator. The design of the CLHLS study was approved by the Campus Institutional Review Board of Duke University (Pro00062871) and the Biomedical Ethics Committee of Peking University (IRB00001052-13074) (29).

### 2.2 Measurements

Demographic variables, including gender, age, ethnicity, household income, residential situation, marital status, number of children, and pension insurance situation, medical insurance situation were gathered from the participants.

The Center for Epidemiologic Studies Depression Scale-10 (CESD-10) is a questionnaire employed for the evaluation of depressive symptoms among participants. In previous studies, scholars have also used the Patient Health Questionnaire-9 (PHQ-9) to measure participants’ depressive conditions (30, 31). However, because the participants of the survey are older adults, the CESD-10 questionnaire has advantages such as being brief and easy to complete, which made it more suitable for older people to answer compared to the PHQ-9 (32, 33). In addition, the CESD-10 has been found to have acceptable internal consistency across samples and adequate concurrent validity, when compared to the PHQ-9 and WHO Disability Assessment Schedule 2.0 (WHODAS) (34). The CESD-10 comprises 10 questions, with answers arranged into four levels: 0 (never), 1 (rarely /sometimes), 2 (often), and 3 (always). The overall score range spans from 0 to 30, with elevated scores indicating an amplified level of depression. According to previous studies, a CESD-10 score ≥ 10 is considered indicative of the presence of depressive symptoms (32).

Anxiety symptoms were assessed by the GAD-7 scale, which measures the frequency of anxiety symptoms in the past 2 weeks. This scale comprises 7 questions, with answers arranged into four levels: 0 (not at all), 1 (a few days), 2 (more than half the days), and 3 (almost every day). Overall, GAD-7 scores range from 0 to 21. When a GAD-7 score exceeded the threshold of 5, it is deemed indicative of the presence of an anxiety disorder (19). Previous studies have shown that the GAD-7 scale has been utilized in network analyses in Chinese populations and has demonstrated great reliability (19).

In the CLHLS project, life satisfaction among hypertensive older adults was measured primarily using the question, “How do you feel about your life now?” (35). Responses to this question were scored on a scale ranging from 1 (very poor) to 5 (very good), with higher scores reflecting elevated levels of LS among participants.

### 2.3 Statistical analyses

All data statistical analyses were conducted using R (Version 4.1.2). The R-package qgraph (version 1.9.2) was used to estimate the network structures and perform visualization of the whole network (14). The present program adopts a Gaussian graphical model (GGM) to investigate ordered or categorical data (36). The regularized casual correlation network is built using the GGM model algorithm, which is based on the glasso (graph minimal absolute shrinkage and control for false positive edges) process (36). Extended Bayesian Information Criterion (EBIC) was used to choose the best-fitting model (19). In a network visualization graph, edges represent the relationship between two nodes after conditioning on all other nodes in the analysis. Positive associations are denoted by blue edges, while negative associations are represented by red edges. The thickness of the lines indicates the strength of the associations. We utilized the mgm package to evaluate the predictability of each node. A node with a high value of predictability indicates that it is explained by its neighboring nodes (37). To explore symptoms of depression and anxiety associated with life satisfaction, we used the “flow” function of the package qgraph, with the advantage of being able to identify focus-related symptoms.

Centrality analysis was used to identify symptoms with high impact (core symptoms) in network structure. In this study, Expected Influence (EI) was used to estimate the centrality symptoms of the network. Previous studies have found that if there is a negative correlation between nodes in a network, applying EI to estimate the network’s centrality symptom will be better than the commonly used “strength” index (38). In addition, the packages botnet and network tools were used to explore bridge symptoms that played important roles in connecting two communities of symptoms (14). This bridge symptom is expressed using the Bridge Expected Influence (BEI) value, where nodes with higher BEI values are more inclined to trigger activation in other communities.

The network accuracy and stability were tested using the R package bootnet (14). First, we bootstrapped the 95% confidence intervals of the edge weights, providing an estimate of the accuracy of edges in the networks. Second, network stability was examined by the case-dropping subset bootstrap function with a correlation stability coefficient (CS coefficient). The CS coefficient should not be below 0.25; a value above 0.50 suggests strong stability and interpretability. Finally, we did edge weights significance tests and centrality difference tests for the network. In the current study, 1,000 bootstrap iterations were used in all cases.

## 3 Results

### 3.1 Study sample

The analysis included a cohort of 4,993 older adults with hypertension in China. The average age was 81.91 (SD = 10.22) years old. Of these participants, 2,195 were male and 2,798 were female. Other demographic variables of the participants are shown in Table 1. Meanwhile, 2,287 participants had significant depressive symptoms (45.80%, scores of ≥10 on the CESD-10) and the mean CESD-10 total score in the participants was 8.75 (SD = 3.91). 612 of these participants had anxiety symptoms (12.3%, scores of ≥5 on the GAD-7) and the mean GAD-7 total score in the participants was 1.42 (SD = 2.71). The mean scores for each item on the CESD-10 and GAD-7 and the predictability for each symptom are shown in Table 2.

**Table 1:** Demographic characteristics of hypertensive older adults.

Variable	N (%)	Mean	SD
Gender		—	—
Male	2,195 (43.99)		
Female	2,798 (56.01)		
Age (year)		81.91	10.22
60 ~ 69 years	632 (12.66)		
70 ~ 79 years	1,534 (30.72)		
80 ~ 89 years	1,547 (30.98)		
≥90 years	1,280 (25.64)		
Ethnic group		—	—
Han	4,350 (87.12)		
Hui	41 (0.82)		
Zhuang	95 (1.90)		
Other	244 (4.89)		
Missing	263 (5.27)		
Annual income (RMB)		51,044.48	37,625.00
≤20,000	1,544 (30.92)		
20,001 ~ 50,000	1,042 (20.87)		
50,001 ~ 100,000	907 (18.17)		
>100,000	1,144 (22.91)		
Missing	356 (7.13)		
Residential situation		—	—
With household member	3,884 (77.79)		
Alone	832 (16.67)		
In a nursing home	218 (4.38)		
Missing	53 (1.06)		
Marital status		—	—
Married	2,426 (48.59)		
Divorced	25 (0.50)		
Widowed	2,451 (49.09)		
Never married	43 (0.86)		
Missing	48 (0.96)		
Number of children		—	—
0	397 (7.95)		
1	443 (8.87)		
2	812 (16.26)		
3	882 (17.66)		
4	818 (16.38)		
≥5	1,345 (26.94)		
Missing	296 (5.93)		
Possession of a retirement pension		—	—
Yes	1,797 (35.99)		
No	3,072 (61.53)		
Missing	123 (2.46)		
Medical insurance for urban workers and residents		—	—
Yes	1,497 (29.98)		
No	3,146 (63.01)		
Missing	350 (7.01)		
The new rural cooperative medical insurance		—	—
Yes	2,659 (53.25)		
No	2,082 (41.70)		
Missing	252 (5.05)		

SD, Standard deviation.

**Table 2:** Descriptive statistics of the CESD-10 and GAD-7 items.

Item abbreviation	Items context	Mean	SD	Predictability
CESD1	Feeling bothered	0.797	0.578	0.396
CESD2	Difficulty with concentrating	1.049	0.702	0.203
CESD3	Feeling blue/depressed	0.734	0.559	0.478
CESD4	Everything was an effort	1.053	0.727	0.305
CESD5	Hopelessness	1.016	0.784	0.208
CESD6	Feeling nervous/fearful	0.656	0.528	0.360
CESD7	Lack of happiness	1.190	0.891	0.227
CESD8	Loneliness	0.689	0.659	0.366
CESD9	Inability to get going	0.520	0.586	0.374
CESD10	Sleep disturbances	1.043	0.635	0.096
GAD1	Nervousness or anxiety	0.312	0.558	0.596
GAD2	Uncontrollable worry	0.218	0.490	0.687
GAD3	Generalized worry	0.257	0.536	0.639
GAD4	Trouble relaxing	0.185	0.464	0.665
GAD5	Restlessness	0.148	0.414	0.601
GAD6	Irritability	0.183	0.448	0.532
GAD7	Fear of awful events	0.121	0.372	0.473

CESD-10: the ten-item Center for Epidemiologic Studies Depression Scale; GAD-7: the seven-item Generalized Anxiety Disorder Scale; SD: Standard deviation.

### 3.2 Network structure

**Figure 1:**
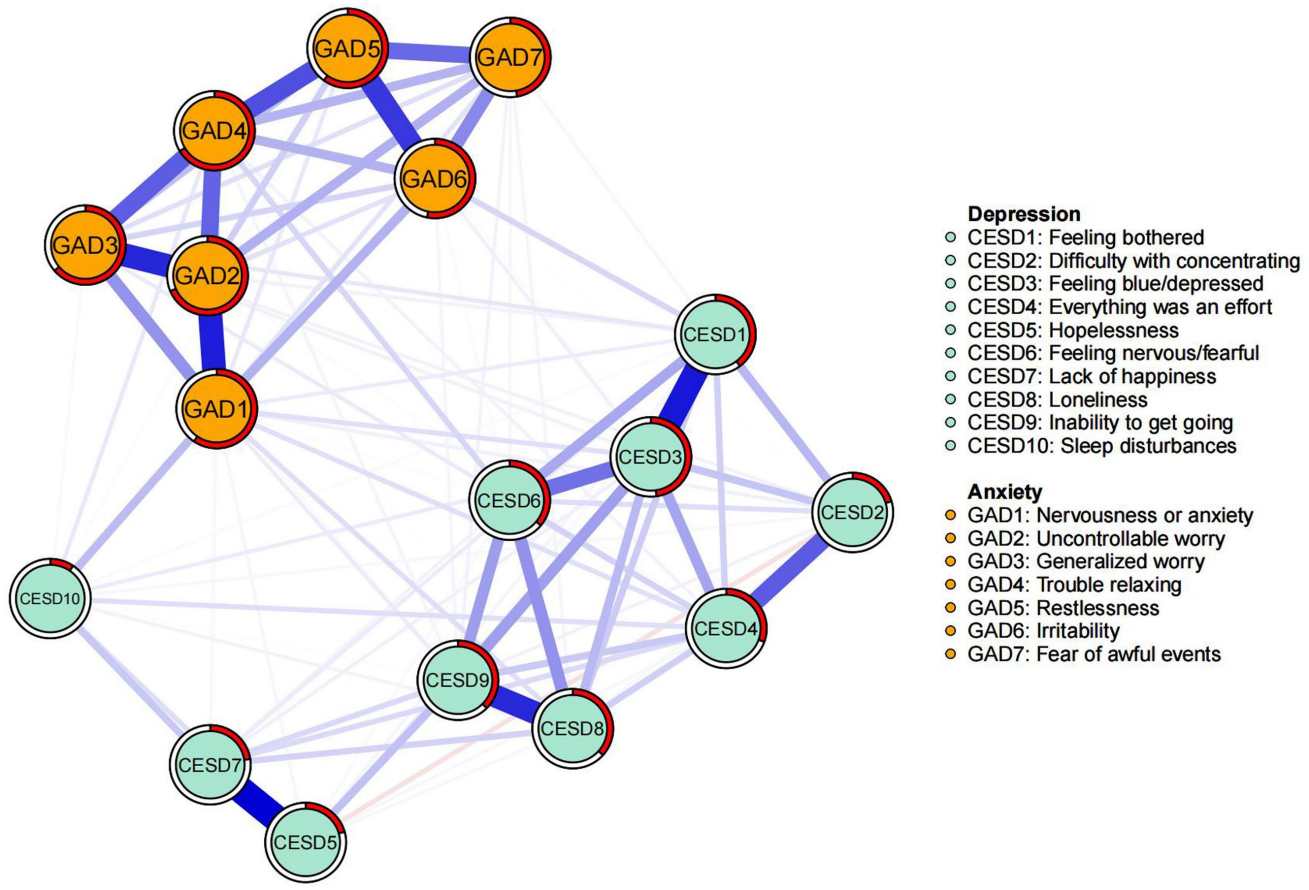
The network structure of depressive and anxiety symptoms in hypertensive older adults.

The network structure of depression and anxiety in Chinese hypertensive older adults was illustrated in Figure 1. Based on the estimated 101 non-zero edges among all possible 136 edges, the network density is approximately 74.26%. The strongest association among depressive symptoms was CESD5 (Hopelessness) and CESD7 (Lack of happiness), followed by an edge for CESD1 (Feeling bothered) and CESD3 (Feeling blue/depressed). The two symptoms with the strongest correlation in the network of anxiety symptoms were GAD1 (Nervousness or anxiety) and GAD2 (Uncontrollable worry), followed by GAD5 (Restlessness) and GAD6 (Irritability). Within the cross-diagnostic network of depression and anxiety, the strongest connection can be found between CESD10 (Sleep disturbances) and GAD1 (Nervousness or anxiety), followed closely by the associations between CESD1 (Feeling bothered) and GAD6 (Feeling nervous/fearful), as well as CESD6 (Feeling nervous/fearful) and GAD4 (Trouble relaxing). Correlation matrices were shown in the Supplementary Table S1.

**Figure 2:**
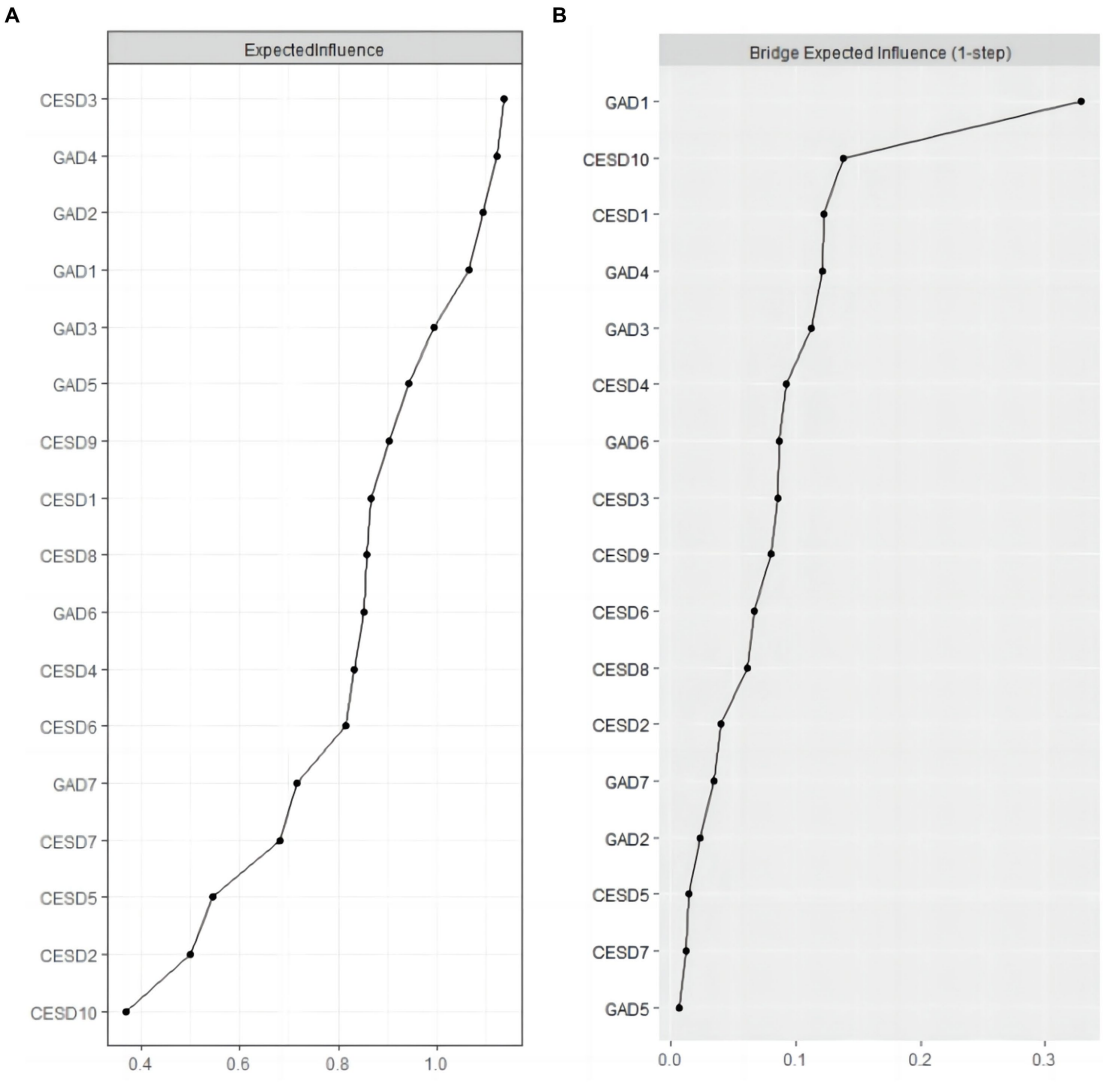
Expected Influence and Bridge Expected Influence of CESD-10 and GAD-7 items. **(A)** illustrates values for Expected influence centrality, and **(B)** illustrates Bridge Expected Influence centrality. Items are listed in descending order from top to bottom, from nodes with the largest centrality value to the smallest centrality value.

The EI and BEI of each node in the depression and anxiety network were shown in Figure 2. CESD3 (Feeling blue/depressed) had the highest expected influence, while GAD4 (Trouble relaxing) and GAD2 (Uncontrollable worry) were also statistically stronger than most other nodes in the depression and anxiety network (Figure 2A). These three symptoms have a significant impact on the network of depression and anxiety symptoms in hypertensive older adults. Additionally, in terms of the BEI analysis, it was found that GAD1 (Nervousness or anxiety), CESD10 (Sleep disturbances), and CESD1 (Feeling bothered) were identified as the central bridge symptoms connecting depression and anxiety (Figure 2B).

### 3.3 Network stability

**Figure 3:**
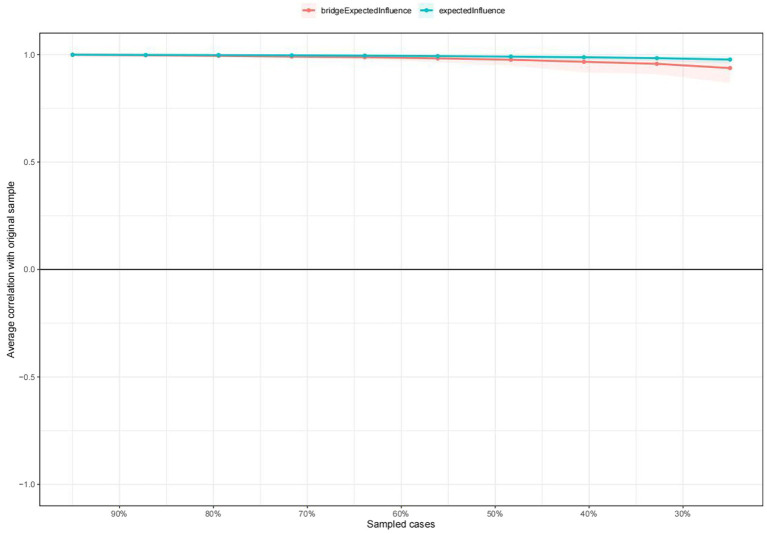
The stability of centrality and bridge centrality indices using case-dropping bootstrap.

The stability of the centrality of EI and BEI was demonstrated to be remarkably high, as indicated by the results presented in Figure 3. The CS coefficient of 0.75 for node EI and bridge EI indicated that when 75% of the sample was dropped, the structure of the network did not significantly change. Supplementary Figure S1 showed that there was considerable overlap among the 95% confidence intervals of edge weights which could be sufficient to indicate that the estimates of the depression and anxiety symptom network were enough accurate. The results of the estimation of edge weight difference by bootstrapped difference test were shown in Supplementary Figure S2. The differences in edge weights have been demonstrated to be statistically significant in most comparisons, as shown by the bootstrap difference test (Supplementary Figure S3).

### 3.4 Flow network of life satisfaction

**Figure 4:**
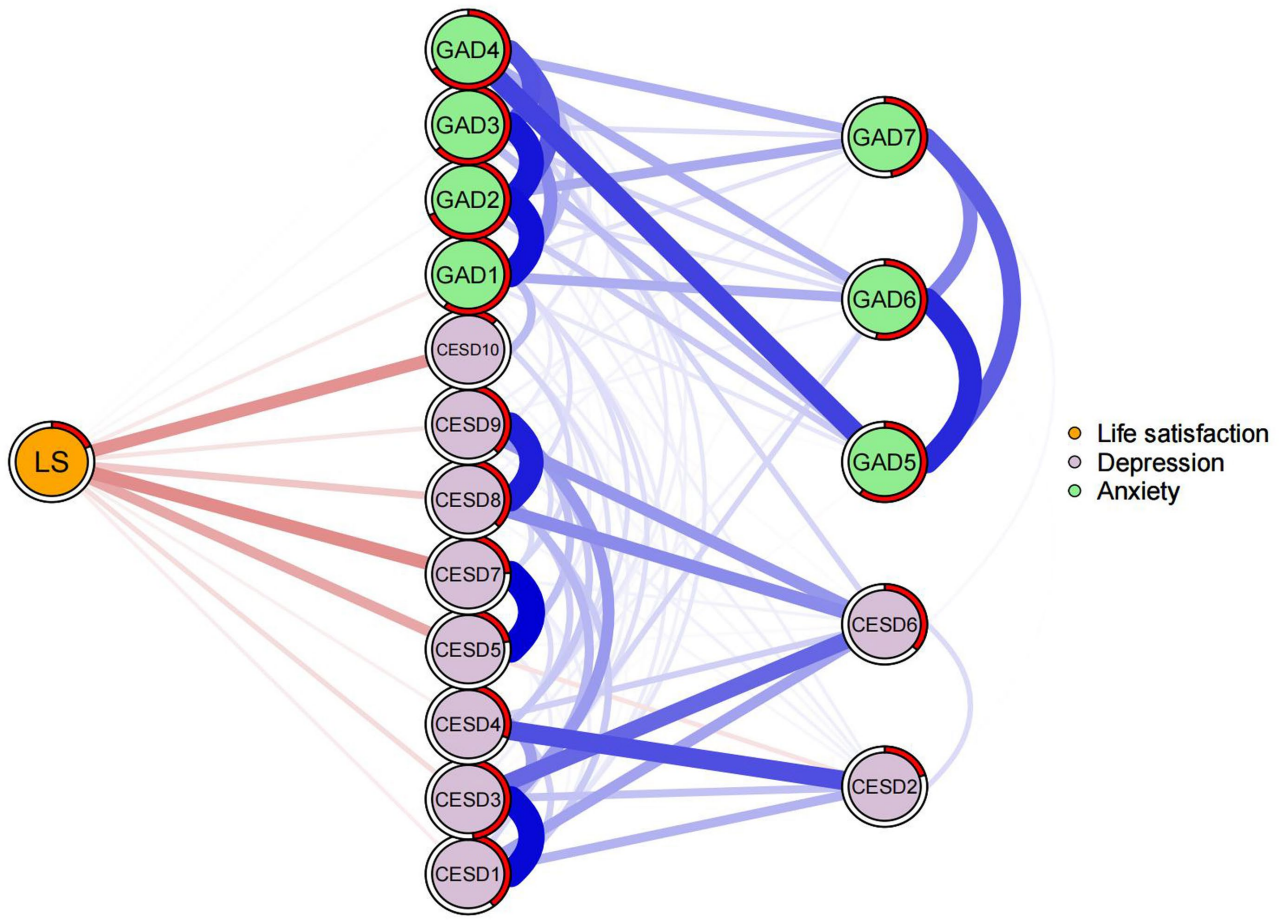
Flow network of life satisfaction.

Figure 4 depicted a flowchart of the network structure of LS in relation to depressive and anxiety symptoms. The 12 nodes located in the middle of the figure are directly related to LS, while the remainder five nodes are indirectly related to LS. Specifically, the node CESD7 (Lack of happiness) had the strongest negative association with LS (average edge weight = −0.152), followed by the CESD10 (Sleep disturbances) (average edge weight = −0.144) and CESD5 (Hopelessness) (average edge weight = −0.115).

## 4 Discussion

A large sample of Chinese hypertensive older adults has been analyzed using network analysis for the first time to examine how depression and anxiety symptoms interact. Within the depression and anxiety network, CESD3 (Feeling blue/depressed), GAD4 (Trouble relaxing), and GAD2 (Uncontrollable worry) emerged as the most influential and central symptoms. Concurrently, we have also discerned that GAD1 (Nervousness or anxiety), CESD10 (Sleep disturbances), and CESD1 (Feeling bothered) stand as pivotal bridge symptoms between depression and anxiety disorders. In addition, the CESD7 (Lack of happiness), CESD10 (Sleep disturbances), and CESD5 (Hopelessness) were negatively associated with LS among Chinese hypertensive older adults.

The results of the network analysis showed strong associations between relationships within depressive symptoms in Chinese hypertensive older adults and the strongest associations were CESD5 (Hopelessness) and CESD7 (Lack of happiness). For anxiety disorder, the strongest associations between anxiety symptoms were GAD1 (Nervousness or anxiety) and GAD2 (Uncontrollable worry). The results of this study are consistent with those of previous articles that examined psychiatric disease network structures (15, 21). Bai’s investigation revealed that among the older adult population, the symptoms of hopelessness and lack of happiness are most strongly interconnected within the network of depressive symptoms (15). A network analysis study on depression and anxiety among vocational nurses after the COVID-19 epidemic outbreak revealed that the strongest association among anxiety symptoms was between nervousness and uncontrollable worry (21). According to the network theory of mental disorders, there exists mutual influence among internal symptoms, whereby the alleviation of one symptom may simultaneously lead to the improvement of another, thereby reducing the overall severity of the disorder (16). A randomized controlled trial (RCT) of the 3 L-Mind-Training program conducted among older adult residents in nursing homes discovered that enhancing the sense of happiness in older adults can alleviate their sense of hopelessness and feelings of depression in long-term care facilities (39). During the initial stages of intervening in depression and anxiety disorders, it is necessary to identify the strongest symptom connections within each ailment using network analysis, then focus our efforts on intervening strongest connected symptoms.

CESD-3 (Feeling blue/depressed) was the most central symptom for Chinese hypertensive older adults among depression-anxiety network, suggesting that it plays the greatest influential role in constructing and sustaining the depression and anxiety psychiatric disorders network. A recent psychiatric network analysis study among Chinese older adults with disabilities found CESD-3 (Feeling blue/depressed) to be the most core symptom among depression-anxiety network (19). Similarly, a network analysis study conducted in Stockholm, Sweden between depressive symptoms and chronic disease clusters in 2,860 aging people over 60 years of age revealed that depressive moods such as sadness and pessimism were the most significant and influential symptoms in the network (40). Although the cultural background, physical status, and illnesses of the subjects of these two network analysis studies were different from our study, they were both studied in older adults and the results were consistent in terms of network centrality. This suggests that there is a commonality of central symptoms in the older adult population and that “Feeling blue/depressed” is the most centrally important symptom. As a consequence of the physiological degradation and escalating afflictions associated with aging, coupled with the gradual loss of social roles, senior individuals become increasingly susceptible to symptoms of sorrow and depression, further increasing the susceptibility to developing depression (41). In addition, the second and third highest ranked centrality symptoms according to the magnitude of the values in the EI were GAD4 (Trouble relaxing) and GAD2 (Uncontrollable worry). However, A network analysis of a large sample of patients diagnosed with depressive-anxiety comorbidity by clinicians before and after hospitalization found that “Uncontrollable worry” was identified as a central symptom, but “Trouble relaxing” was not identified as a central symptom (22). This discrepancy in findings can be attributed to the unique characteristics of the sample population. Different patient groups may exhibit variations in their symptom profiles of disease and symptoms network analysis, which can contribute to the variability in identifying central symptoms. This emphasizes the importance of considering the specific context and symptom characteristics of the studied population when interpreting central symptoms in network analysis (23).

In the construction and sustainment of psychiatric co-morbidity networks, the magnitude of BEI values corresponding to individual symptoms reflects their bridge centrality and critical bridge symptoms contribute significantly to the maintenance of the two community networks. In our study, GAD1 (Nervousness or anxiety) was a critical bridge symptom in the current depression-anxiety network in the Chinese hypertensive older adults, which was consistent with previous findings (19, 42). Separate network analysis studies of older adults during the COVID-19 epidemic (42) and a Chinese population of older adults with disabilities (19) confirmed that GAD1 is the most critical bridge symptom connecting the two communities of depression and anxiety (19, 42). However, the results were not the same as the core bridge symptom results from the adolescent and adult depression-anxiety network analysis studies (20, 43). The central bridge symptoms of depression and anxiety network may vary among different age demographics, requiring target-specific analysis of samples with distinct characteristics. A population-based cohort study in Sweden found that people with hypertension had higher nervousness scores compared to normal people (44). To our knowledge, older adults with hypertension are inherently considered a vulnerable population, and the presence of physical complications from hypertension and concerns about future life can exacerbate their feelings of nervousness (5), which may eventually lead to depression, anxiety, and other psychological health issues (6). In addition, both exploratory factor analysis studies and network analysis studies have discovered that nervousness is an important transdiagnostic symptom associated not only with anxiety but also with depression (19, 42, 45). In the present study, CESD-10 (Sleep disturbances) and CESD1 (Feeling bothered) were also central bridge symptoms. Sleep disturbances are closely linked with hypertension. The nocturnal hyperactivity of the sympathetic nervous system in individuals with hypertension can disrupt regular sleep patterns, and elevate heart rate and blood pressure, thereby mitigating the quality of slumber and precipitating sleep disturbances such as insomnia or sleep apnea (46–48). Sleep disturbances may impair emotional regulation and stress response mechanisms, perpetuating a cycle of negative emotions, and could exacerbate or precipitate symptoms of depressive and anxiety disorders (49, 50). Nevertheless, few investigations have identified CESD1 (Feeling bothered) as the central bridge symptoms for anxiety and depressive network. This requires us to pay attention to the symptom of “Feeling bothered” in future studies of depression and anxiety among hypertensive older adults. In-depth investigation into the mechanisms by which “Feeling bothered” acts as a bridge symptom could provide valuable guidance for the development of treatments targeting comorbid depression and anxiety.

Previous studies have already established a negative association between individuals’ encounters with depression and anxiety disorders and their levels of life satisfaction (27, 28). In this study, we focused on which specific symptoms of depression and anxiety disorders are directly related to life satisfaction. Compared with other symptoms, CESD7 (Lack of happiness) had the strongest negative associations with LS in hypertensive older adults. The negative association between lack of happiness and life satisfaction can be better understood through psychological and sociological explanations. On the one hand, the psychological argument holds that individuals lacking a sense of happiness may tend to focus on negative experiences and negative emotions in their lives, thereby diminishing their overall satisfaction in various aspects of life (51, 52). Conversely, positive emotions can significantly enhance psychological functioning and happiness (53), contributing to individual life satisfaction. On the other hand, the sociological perspective’s explanation emphasizes the influence of social environment and social interactions on happiness and life satisfaction (54). Individuals lacking a sense of happiness may find themselves experiencing social isolation, feelings of loneliness, or a lack of social support, all of which are closely associated with a decrease in life satisfaction (55, 56).

This study utilized a large dataset of older adults with hypertension to conduct a network analysis from the perspective of specific symptoms of depression, anxiety, and life satisfaction. By exploring the cross-diagnostic connections among these specific symptoms, the research provided theoretical underpinnings for the intervention of mental health issues such as depression and anxiety in hypertensive older adults. Although this study had advantages such as a large sample size and representative sample population, there were still some potential limitations. First, because of the cross-sectional character of the data, the network analysis is undirected, and causal inference may be limited. Second, because this study investigated sites in the communities rather than hospitals, information on the impact of comorbidities and clinical substance use on participants’ mental health was not included, and the fact that these two potential factors were not controlled for is one of the limitations of this study. Third, during the selection of subjects for our study, we specifically included older hypertensive patients but did not consider whether they had a history of psychiatric illness, which may introduce a relevant bias. Future studies may investigate hypertensive older adults with various subtypes of psychiatric history in order to construct distinct symptom networks. Forth, Previous researches have already demonstrated that sleep (57, 58) and physical activity (59, 60) are very important factors that have significant impacts on an individual’s physical and mental health. However, this study has not yet considered the effects of sleep and physical activity on mental disorders such as depression and anxiety in older adults with hypertension. Last but not least, it should be noted that the present study findings are limited in their generalizability beyond the specific population of older adults with hypertension. Caution must be exercised when attempting to apply these results to other cohorts of older adults with different chronic diseases.

## 5 Conclusion

In conclusion, this exploratory study represents the first investigation to uncover the mutual relationship between depressive symptoms and anxiety symptoms among older adults with hypertension in China. Interventions targeting bridge symptoms such as GAD1 (Nervousness or anxiety), CESD10 (Sleep disturbances), and CESD1 (Feeling bothered) have the potential to alleviate depressive and anxiety symptoms in this population. Furthermore, improving happiness, hope, and sleep quality in older adults with hypertension may reduce the negative impact of depression and anxiety on LS.

## Data availability statement

Publicly available datasets were analyzed in this study. This data can be found here: The data used in this research comes from the Chinese Longitudinal Healthy Longevity Survey (CLHLS Program), which requires authorization from the Peking University Open Research Data Platform before it can be utilized.

## Ethics statement

During the field survey, all hypertensive older adults signed an informed consent form under the direction of the investigator. The design of the CLHLS study was approved by the Campus Institutional Review Board of Duke University (Pro00062871) and the Biomedical Ethics Committee of Peking University (IRB00001052-13074).

## Author contributions

HM: Data curation, Formal analysis, Investigation, Methodology, Project administration, Resources, Software, Validation, Visualization, Writing – original draft, Writing – review & editing. MZ: Data curation, Formal analysis, Investigation, Software, Writing – review & editing. YL: Conceptualization, Methodology, Writing – review & editing. PW: Conceptualization, Funding acquisition, Software, Supervision, Validation, Writing – review & editing.
