# Supplementary material for: Network analysis of depression and anxiety symptoms and their associations with life satisfaction among Chinese hypertensive older adults: a cross-sectional study
Source: Front Public Health. 2024 Mar 18;12:1370359. doi: 10.3389/fpubh.2024.1370359 (PMC10983850; doi:10.3389/fpubh.2024.1370359)

Supplementary Materials

For

“Network analysis of depression and anxiety symptoms and their associations with life satisfaction among Chinese hypertensive older adults: A cross-sectional study”

|  | **CESD.1** | **CESD.2** | **CESD.3** | **CESD.4** | **CESD.5** | **CESD.6** | **CESD.7** | **CESD.8** | **CESD.9** | **CESD.10** | **GAD.1** | **GAD.2** | **GAD.3** | **GAD.4** | **GAD.5** | **GAD.6** | **GAD.7** |
| --- | --- | --- | --- | --- | --- | --- | --- | --- | --- | --- | --- | --- | --- | --- | --- | --- | --- |
| **CESD.1** | 0.000 |  |  |  |  |  |  |  |  |  |  |  |  |  |  |  |  |
| **CESD.2** | 0.101 | 0.000 |  |  |  |  |  |  |  |  |  |  |  |  |  |  |  |
| **CESD.3** | **0.329** | 0.081 | 0.000 |  |  |  |  |  |  |  |  |  |  |  |  |  |  |
| **CESD.4** | 0.065 | **0.233** | 0.127 | 0.000 |  |  |  |  |  |  |  |  |  |  |  |  |  |
| **CESD.5** | 0.013 | -0.042 | 0.031 | 0.012 | 0.000 |  |  |  |  |  |  |  |  |  |  |  |  |
| **CESD.6** | 0.122 | 0.047 | 0.201 | 0.062 | 0.008 | 0.000 |  |  |  |  |  |  |  |  |  |  |  |
| **CESD.7** | 0.027 | 0.000 | 0.019 | 0.052 | **0.362** | 0.016 | 0.000 |  |  |  |  |  |  |  |  |  |  |
| **CESD.8** | 0.073 | 0.011 | 0.099 | 0.068 | 0.012 | 0.153 | 0.062 | 0.000 |  |  |  |  |  |  |  |  |  |
| **CESD.9** | 0.008 | 0.018 | 0.135 | 0.075 | 0.089 | 0.137 | 0.054 | **0.303** | 0.000 |  |  |  |  |  |  |  |  |
| **CESD.10** | 0.006 | 0.011 | 0.027 | 0.046 | 0.046 | 0.000 | 0.077 | 0.017 | 0.003 | 0.000 |  |  |  |  |  |  |  |
| **GAD.1** | 0.027 | 0.020 | 0.044 | 0.044 | 0.013 | 0.005 | 0.009 | 0.045 | 0.027 | **0.094** | 0.000 |  |  |  |  |  |  |
| **GAD.2** | 0.018 | 0.000 | 0.000 | 0.004 | 0.000 | 0.000 | 0.000 | 0.002 | 0.000 | 0.000 | **0.319** | 0.000 |  |  |  |  |  |
| **GAD.3** | 0.033 | 0.018 | 0.019 | 0.028 | 0.000 | 0.000 | 0.000 | 0.000 | 0.008 | 0.007 | 0.152 | 0.308 | 0.000 |  |  |  |  |
| **GAD.4** | 0.000 | 0.000 | 0.000 | 0.017 | 0.000 | **0.057** | 0.003 | 0.000 | 0.014 | 0.031 | 0.087 | **0.221** | **0.228** | 0.000 |  |  |  |
| **GAD.5** | 0.000 | 0.000 | 0.004 | 0.000 | 0.000 | 0.000 | 0.000 | 0.000 | 0.000 | 0.004 | 0.032 | 0.068 | 0.090 | **0.250** | 0.000 |  |  |
| **GAD.6** | **0.057** | 0.002 | 0.019 | 0.000 | 0.000 | -0.006 | 0.000 | 0.000 | 0.014 | 0.000 | 0.109 | 0.042 | 0.059 | 0.108 | **0.283** | 0.000 |  |
| **GAD.7** | -0.013 | 0.000 | 0.000 | 0.000 | 0.001 | 0.013 | 0.000 | 0.014 | 0.018 | 0.002 | 0.039 | 0.112 | 0.044 | 0.106 | **0.213** | 0.166 | 0.000 |

Table S1. Correlation matrix of the CESD-10 and GAD-7 items

Fig. S1. Bootstrapped confidence intervals of edge weights


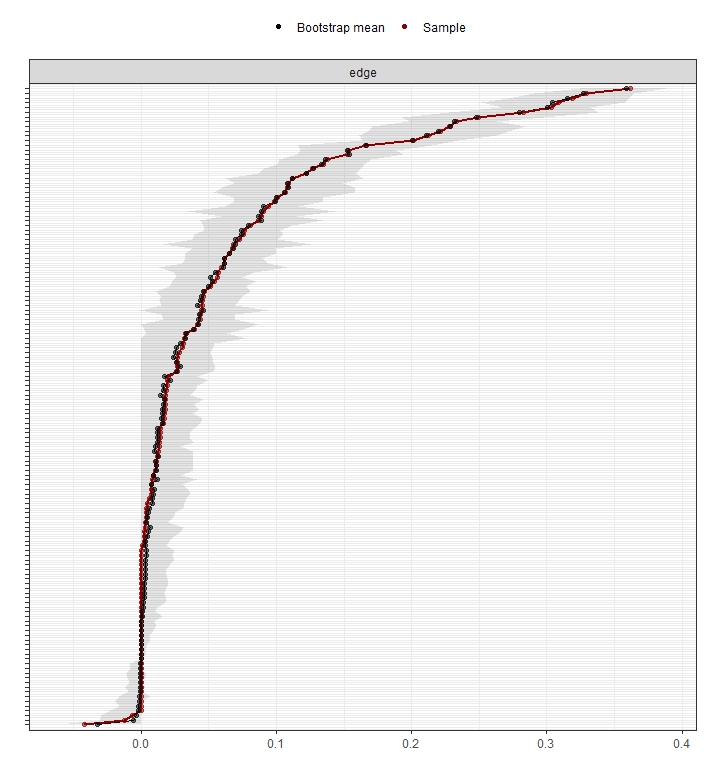


Fig. S2. Estimation of edge weight difference by bootstrapped difference test


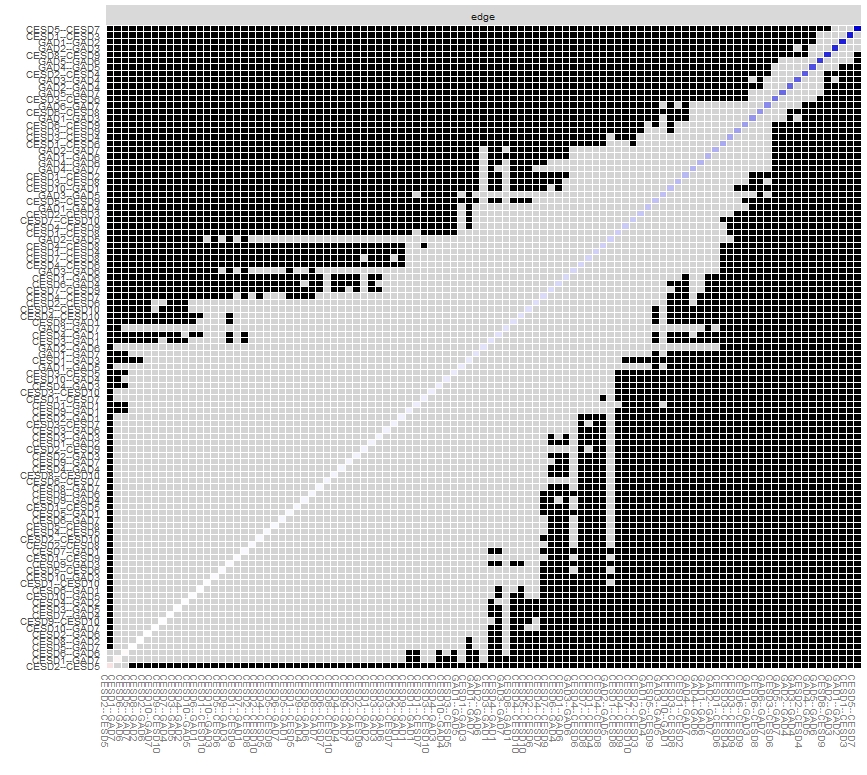


Fig. S3. Nonparametric bootstrapped difference test


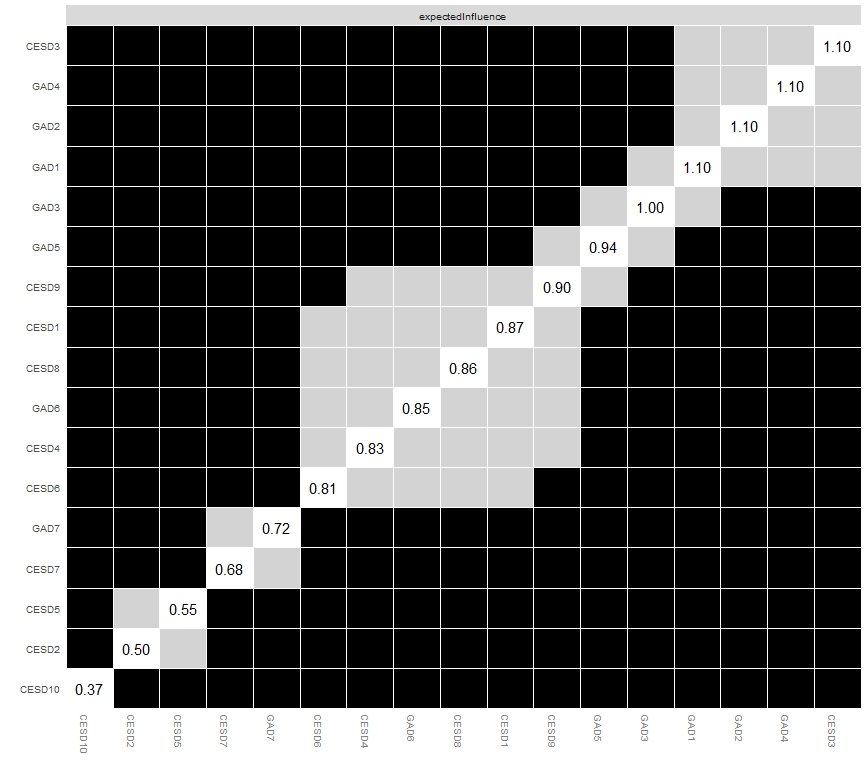

Supplement: Supplementary file 1 [file Data_Sheet_1.docx]
